# Long-term safety of rituximab induced peripheral B-cell depletion in autoimmune neurological diseases

**DOI:** 10.1371/journal.pone.0190425

**Published:** 2018-01-08

**Authors:** Anza B. Memon, Adil Javed, Christina Caon, Shitiz Srivastawa, Fen Bao, Evanthia Bernitsas, Jessica Chorostecki, Alexandros Tselis, Navid Seraji-Bozorgzad, Omar Khan

**Affiliations:** 1 Multiple Sclerosis Center, Department of Neurology, Wayne State University School of Medicine, Detroit, Michigan, United States of America; 2 Multiple Sclerosis Center, Department of Neurology, University of Chicago Pritzker School of Medicine, Chicago, Illinois, United States of America; 3 The Sastry Foundation Advanced Imaging Laboratory, Department of Neurology, Wayne State University School of Medicine, Detroit, Michigan, United States of America; Klinikum rechts der Isar der Technischen Universitat Munchen, GERMANY

## Abstract

**Background:**

B-cells play a pivotal role in several autoimmune diseases, including patients with immune-mediated neurological disorders (PIMND), such as neuromyelitis optica (NMO), multiple sclerosis (MS), and myasthenia gravis (MG). Targeting B-cells has been an effective approach in ameliorating both central and peripheral autoimmune diseases. However, there is a paucity of literature on the safety of continuous B-cell depletion over a long period of time.

**Objective:**

The aim of this study was to examine the long-term safety, incidence of infections, and malignancies in subjects receiving continuous therapy with a B-cell depleting agent rituximab over at least 3 years or longer.

**Methods:**

This was a retrospective study involving PIMND who received continuous cycles of rituximab infusions every 6 to 9 months for up to 7 years. The incidence of infection related adverse events (AE), serious adverse events (SAE), and malignancies were observed.

**Results:**

There were a total of 32 AE and 4 SAE with rituximab treatment. The 3 SAE were noted after 9 cycles (48 months) and 1 SAE was observed after 11 cycles (60 months) of rituximab. There were no cases of Progressive multifocal leukoencephalopathy (PML) and malignancies observed throughout the treatment period. Rituximab was well tolerated without any serious infusion reactions. Also, rituximab was found to be beneficial in treating PIMND over a 7-year period.

**Conclusions:**

This study demonstrates that long-term depletion of peripheral B-cells appears safe and efficacious in treating PIMND. Longer and larger prospective studies with rituximab are needed to carefully ascertain risks associated with chronic B-cell depletion, including malignancies. Recognizing that this is a small, retrospective study, such data nonetheless complement the growing literature documenting the safety and tolerability of B-cell depleting agents in neurological diseases.

## 1. Introduction

B-cells play an important role in diverse autoimmune diseases, including neurological, connective tissue, and vasculitic disorders. They are involved in antigen presentation, epitope specific autoantibody production, and cytokine production [1]. Given their central role in generating autoantibodies, they have become an important target for several autoimmune diseases.

Rituximab is a human-mouse monoclonal chimeric antibody that targets CD20 molecules, which are expressed by B-cells during their maturation. CD20 is a cell surface antigen, which is expressed on pre-B cells, mature B cells and memory B cells [2]

CD20 is not expressed by hematopoietic stem cells and pro-B cells, and is subsequently lost upon terminal differentiation into plasma cells [3]. Antagonism of CD20 by rituximab does not prevent B-cell regeneration nor does it affect plasmablast or plasma cell differentiation [4]. Rituximab causes short-term depletion of circulating naïve and memory B-cells through three main mechanisms: antibody-dependent cell mediated cytotoxicity, complement mediated cytotoxicity, and induction of apoptosis [5–7]. Tissue levels of CD20 expressing cells may be affected to a lesser degree than circulating CD20 cells [8–9]. Also, repletion rate of circulating and tissue levels of CD20 B-cells are variable [8–9].

Rituximab has been successfully used in the treatment of several diseases driven by B-cell dysregulation. In the U.S., it is approved for the treatment of various B-cell driven malignancies, certain forms of vasculidites, and rheumatoid arthritis. Rituximab is often used as an “off-label” therapy in patients with immune-mediated neurological disorders (PIMND) including multiple sclerosis, autoimmune neuropathies, neuromyelitis optica, myasthenia gravis, paraneoplastic neurological disorders, and inflammatory myopathies [10–12]. Given the promising results of several open-label and randomize controlled studies of rituximab in various disorders, there is considerable interest in further development of B-cell depleting or B-cell anti-proliferative agents. The enthusiasm over the promising results is curtailed by concerns about the long-term safety of rituximab or other similar therapies. This risk is amplified because chronic administration of therapies for PIMND is often for several years if not life-long.

Recognizing the rapidly evolving therapeutic development of B-cell depleting therapies and the growing interest of the neurological community, we investigated the long-term safety of continuous use of rituximab in PIMND that included patients with MS, NMO, and MG.

## 2. Methods

### 2.1 Study population

This was a retrospective study conducted at two tertiary centers involving patients with the diagnosis of MS, NMO, and MG, involving a chart-review of patients who had received rituximab from 2008–2014 for at least 36 months continuously without any interruption. The local Institutional Review Boards approved the study.

### 2.2 Treatment protocol

Rituximab was administered intravenously every 6 months (site 1, Detroit, MI) or 6-9-month cycle depending on the level of circulating CD19 blood count (site 2, Chicago, IL), which was always < 5% of lower limit of normal range. The initial course of rituximab comprised of 1000 mg administered intravenously (IV) about 15 days apart. Further infusions were given at 1000 mg IV repeated every 6 to 9 months. All patients were pretreated with methylprednisolone 500–1000 mg IV, diphenhydramine 50 mg IV, and acetaminophen 650 mg orally prior to each rituximab infusion. Peripheral blood B-cell counts (using CD19 expression) were obtained at baseline and prior to each treatment cycle. Patients were seen in the clinic for neurological examination and safety evaluation about every 3 months.

### 2.3 Analysis

Primary objective of this study was the long-term safety of rituximab in PIMND, who had received at least 3 years of uninterrupted treatment with rituximab. For safety assessments, the occurrence of infections, malignancies, or any unexpected side effects were documented. All infections were noted as adverse event (AE) or serious AE (SAE), if they required hospitalization. Infections and malignancies were self-reported by the patients and documented in charts.

## 3. Results

In this retrospective study, 29 patients were identified as having received rituximab continuously for at least 36 months (22 women and 7 men). The mean age of patients was 37.4 ± 10.5 years and included patients with MS (n = 5), MG (3), and NMO (n = 21). Patients demographic details, treatment duration, number of treatment cycles, reported AE, SAE and malignancy are described in Table 1

**Table 1:** Summary of demographical data, rituximab treatments, and adverse events.

Rituximab Group (n = 29)			
Characteristics	NMO (n = 21)	MS (n = 5)	MG (n = 3)
Mean age (37)	35	44.4	41
F:M (22:7)	17:4	3:2	2:1
CD 19 count*	0	0	0
No of cycles†			
5 (n = 2)	2		
7 (n = 5)	5	1	1
8 (n = 4)	2	2	1
9 (n = 4)	4	1	0
11 (n = 7)	6	0	1
13 (n = 3)	2	1	0
Treatment Duration			
36mths (n = 5)	4	1	1
42mths (n = 4)	1	2	1
48mths (n = 4)	6	1	0
60mths (n = 7)	7	0	1
72mths (n = 3)	3	1	0
No of Infections (AE) (n = 32)	25	5	2
No of Infections (SAE) (n = 4)	4	0	0
Malignancy (n = 0)	0	0	0

* At site 1, counts obtained every 6 months post-rituximab therapy (N = 23). At site 2, the C19 count from 6–9 months was always < 5% of the lower limit of normal range

†Cycles are given every 6–9 months

The mean duration of treatment with rituximab was 51.3 ± 12.2 months. The mean number of rituximab treatment cycles was 8.83 ± 2.85. In most patients (site 1, n = 23), the number of CD 19 count at month 12 and every 6-month post rituximab therapy remained zero throughout the treatment period from 36–72 months. In a smaller cohort (site 2, n = 6), the CD19 count was always less than 5% of the lower limit of normal range.

There were total of 32 AE reported in patients. There were total of 4 SAEs noted in this study (Tables 1 and 2). The 3 SAE were noted after 9 cycles of rituximab (48 months) and 1 SAE was observed after 11 cycles of rituximab (60 months). There were no cases of progressive multifocal leukoencephalopathy (PML) or malignancies observed throughout the observation period. Also, repeated rituximab infusions were well tolerated over time without any significant infusion reactions.

**Table 2:** Comparison of adverse events between the rituximab treated group and the control group.

Groups	No. Infections (pts)	Details	Infection rate (SD)
Rituximab (n = 29)			
Infections (AE)	32 (19)	URI, UTI, cellulitis	1.1 ± 1.0
Serious Infections (SAE)	4 (3)	pneumonia, UTI, sinusitis	0.1 ± 0.4

Upper respiratory infection, URI; urinary tract infection, UTI; SAE (Required IV antibiotics or hospitalization); patients, pts; standard deviation, SD.

The rate of AE and SAE observed in the PIMND group treated with rituximab over a 7-year observation period remained low.

Although this study was designed primarily to assess the safety of rituximab in PIMND over a long period of time and not necessarily the efficacy of rituximab in this patient population, nevertheless, an overall benefit of rituximab in terms of relapse rate reduction and improvement in expanded disability status scale (EDSS) was observed in NMO and MS patients from the baseline over a 7-year period.

## 4. Discussion

This study describes the safety of peripheral B-cell depletion using rituximab in PIMND over a relatively longer period of time than has been observed in phase II studies of rituximab in MS [10,13]. The primary objective of the study was to measure the AE and SAE in patients treated with rituximab for at least 3 and up to 7 years. We observed complete depletion of circulating B-cells in the majority of the patients and marked depletion in some of the patients throughout the treatment period. A total of 32 AE and 4 SAE (n = 3) were reported. The mean rate of SAE was 0.1 ± 0.4. All AE were not dose-related and developed regardless of duration and number of treatment cycles with rituximab. The results of this study indicate that the rate and types of AE and SAE were low in patients treated with rituximab. There were no cases of PML or malignancies observed. Rituximab cycles were well tolerated with minimal and manageable infusion related reactions.

Previous studies have also demonstrated relative safety of rituximab albeit over the course of short-term clinical trials. In the Phase II trial of rituximab in relapsing remitting MS patients (RRMS) (48 weeks) the incidence of any infection was similar in the rituximab (69.6%) and placebo (71.4%) groups [10]. SAEs were observed in 13% of rituximab group and 14.3% placebo group. The most common infections reported in this trial were nasopharyngitis, upper respiratory infections, sinusitis, and urinary tract infections. Serious opportunistic infections were not reported. The longer trial of rituximab in primary progressive MS (96 weeks) did show a relatively higher risk of serious infections (4.5%) compare to placebo (<1%) [13]. Most of the serious AE were recorded in patients >55 years of age. The cases of PML associated with rituximab have been reported in patients with lymphoma, rheumatoid arthritis (RA) and lupus who also received multiple immunosuppressive therapies [14–16]. Global clinical trial program of pooled observed case analysis of data on patients (n = 3194) with moderate to severe rheumatoid arthritis treated with rituximab (2x 1000 mg or 2x 500 mg given 2 weeks apart) over 9.5 years demonstrated SAE rate of 3.94/100 patient-years which was comparable to methotrexate + placebo group (3.79/100 patient-years). No opportunistic infections or malignancy were observed. No case of PML was reported [17]. Cases of PML have not been reported in MS patients treated with rituximab. One patient was reported to have malignant thyroid neoplasm in RRMS phase II trial with rituximab [10]. In a retrospective analysis of 25 NMO cases treated with rituximab for a median follow up of 19 months, the incidence of infections was low, fungal infection (n = 1), herpes zoster (n = 1), recurrent clostridium difficile colitis (n = 1) and septicemia (n = 1) [11]. In this study 2 deaths were reported, complicated by recurrent C. difficile colitis and urinary tract related septicemia [11].

In a retrospective, multicenter study involving pediatric patients with autoimmune inflammatory CNS disorders, rituximab was shown to benefit 87% of the patients, with infection AE recorded at 7.6% over a median follow up of 1.65 years [18]. This study included patients with NMDA receptor encephalitis (n = 39), opsoclonus myoclonus ataxia syndrome (n = 32), neuromyelitis optica spectrum disorders (n = 20), neuropsychiatric systemic lupus erythematosis (n = 18) and other neuroinflammatory disorders (n = 35). AE included 2 deaths (grade 5) from cytomegalovirus colitis and staphyloccocal toxic shock syndrome, disabling disease (grade 4) from cytomegalovirus retinitis, shock and hypoxic brain injury (n = 2), and grade 3 infections including pneumonia (n = 2), empyema (n = 1), bronchiectasis (n = 1), salmonella enteritis (n = 1), clostridium difficile enteritis (n = 1) and mastoiditis (n = 1). There were no cases of PML and malignancies reported in this study. Infusion related reactions were seen in 18/144 (12.5%) patients with grade 4 anaphylaxis in 3 patients.

Ofatumumab, another fully humanized monoclonal antibody against CD20, has been approved by the FDA for the treatment of refractory chronic lymphocytic leukemia (CLL). In a randomized double-blinded placebo-controlled trial of ofatumumab in MS, subjects (n = 38) were randomized to 3 doses (100mg, 300mg or 700mg) or placebo 2 weeks apart during the first treatment period (0–24 weeks). Patients then received alternative treatment in the second treatment period (24–48 week) [19]. This study did not reveal any unexpected opportunistic infections and the infusions were generally well tolerated. Albeit a short study, SAE were reported in one patient with placebo (influenza) and 2 patients with ofatumumab (300 mg group). One patient was hospitalized for headache (considered unrelated to ofatumumab) during the 1st treatment period and second patient had prolonged menstrual bleeding leading to anemia during the 2nd treatment period. On the first day of drug administration, infusion related reactions were seen in 2 patients (300 mg group) with grade 3 (pruritic rash, bronchospasm and cough) during the first treatment period and grade 2 (pharyngeal edema, erythema, nasal congestion and pruritis) during the second period.

Recently, the food and drug administration (FDA) approved ocrelizumab for relapsing multiple sclerosis (RMS) and primary progressive multiple sclerosis (PPMS). Ocrelizumab, a fully humanized monoclonal antibody against CD20, causes B-cell depletion similar to rituximab. In the two phase 3 trials of ocrelizumab in RRMS (OPERA I and OPERA II), AE were seen in 80.1% and 86.3% of patients versus 80.9% and 85.6%of patients treated with Rebif [2]. SAEs were reported in 6.9% (OPERA I) and 7% (OPERA II) of patients treated with ocrelizumab versus 7.8% and 9.6% in those treated with Rebif. One death in OPERA II trial was recorded (suicide). Infections were reported to be 56.9% (OPERA I) and 60.2% (OPERA II) of patients in ocrelizumab group vs 54.3% and 52.5% in the Rebif group. Most commonly reported infections were upper respiratory tract infections (15.2%), nasopharyngitis (14.8%) and urinary tract infections (11.6%). Herpes virus associated infection were seen in 5.9% of patients versus 3.4% in the Rebif group. In both OPERA I and OPERA II trials infections were graded as mild or moderate with exception of one case of severe genital herpes (OPERA I) who was treated for 1.6 years and required hospitalization. Infusion related reaction were 34.3% (OPERA I) and 30.9% (OPERA II). One patient in OPERA I trial had life threatening bronchospasm. Most frequent infusion related reactions were reported to be pruritic, throat irritation, flushing and rash [2]. Four neoplasms (0.5% of patients) occurred in Ocrelizumab group (2 cases of invasive ductal breast carcinoma, one case of renal cell carcinoma and one case of malignant melanoma) versus 2 cases (mental cell lymphoma and squamous cell carcinoma in the chest) in the Rebif group (0.2% of patients). Five additional cases of neoplasm were reported during open label extension study with Ocrelizumab (2 breast cancer, 2 basal cell skin cancer, 1 malignant melanoma). In patients with multiple sclerosis who were treated with ocrelizumab, the overall incidence rate of first neoplasm was 0.40/100 patient-years of exposure to ocrelizumab versus 0.20/100 in comparator groups (Rebif or placebo).

In phase 3 trial of ocrelizumab compared to placebo in primary progressive multiple sclerosis (ORATORIO), infusion related reactions (39.9% vs 25.5%), upper respiratory tract infections (11.5% vs 5.9%), oral herpes infections (2.3% vs 0.4%) were higher in ocrelizumab group. 11/486 (2.3% vs 0.8% placebo group) were diagnosed with neoplasms (4 breast cancers, 3 basal cell carcinomas, 1 endometrial adenocarcinoma, 1 anaplastic large T cell lymphoma, 1 malignant fibrous histiocytoma, 1 pancreatic carcinoma). Total of four deaths were reported in the ocrelizumab group (0.8% vs 0.4% in placebo), comprising of pulmonary embolism, pneumonia, pancreatic carcinoma, aspiration pneumonia.

In contrast to the OPERA and ORATORIO trials of Ocrelizumab in MS, our study although with a small sample size but with a much longer duration showed low incidence of adverse events. Prolonged rituximab induced depletion of B-cell did not lead to any life threatening SAEs including malignancy. Safety results of our study are further supported by other studies. One study involved patients with neuromyelitis optica spectrum disorder (n = 30) who received treatment with rituximab for a median of 60 months [20]. In this study, two different regimens of rituximab induction were used, 375 mg /m2 infused once weekly for 4 weeks (n = 16) and 1000 mg infused twice at a 2 weeks interval (n = 14), followed by maintenance therapy depending on the emergence of memory B-cells. No cases of PML or malignancy, SAEs requiring hospitalization, or discontinuation of therapy were reported. In this study 40% of patients had infusion related adverse events during first year of treatment period. No mention of pretreatment prior to rituximab infusion is discussed in the study, which was not the case in our study group where almost all patients received pretreatment with methylprednisolone (500–1000 mg IV) diphenhydramine (50 mg IV) and acetaminophen (650 mg po). A large retrospective observational study investigated the efficacy and safety of rituximab in MS patients (557 RRMS, 198 SPMS and 67 PPMS) [21]. Mean follow up for this study was 21.8 months. A total of 89 AEs grades > 2 (76 infections) were recorded in 72 patients only. No case of PML was reported despite positive JC virus status in 83.3% of patients. Only two grade 3 malignancies were recorded (2 basalioma and 1 pyoderma gangrenosum). No breast malignancies were reported. Mild infusions reactions (headache, chills, nausea and malaise) were observed in 7.8% of infusions, commonly seen during first 3 infusions. There were 4 deaths in the study, which were unrelated to rituximab.

Ocrelizumab has also been studied in Phase III trials in lupus erythematosus and rheumatoid arthritis [22–23]. Both of these Phase III trials showed a higher rate of SAE including grade 5. However, it should be noted that patients in these trials were being concomitantly treated with other immunosuppressive agents along with ocrelizumab. Conversely, in a Phase II trial of ocrelizumab in MS involving patients without prior exposure or concomitant use of any immunosuppressive agent, serious infections were reported at a similar rate in the ocrelizumab and placebo groups [24]. Infusion related adverse events occurred in patients receiving the higher dose, 2000 mg (44%) compared to the group with 600 mg (35%) and placebo (9%). Once death was reported in a 41-year-old patient with MS receiving a 2000 mg dose who died from systemic inflammatory reaction syndrome at week 14.

When rituximab and ocrelizumab are compared to each other in terms of efficacy, safety, and tolerability, albeit without head-to-head comparison, important differences between the two molecules appear to emerge, which may have clinical relevance. Most of the experience with rituximab is based on phase II trials or retrospective data, particularly in MS disease state. The side effect profile of rituximab and ocrelizumab seen in the trials involving RA and SLE patients may not be relevant to PIMND because of the differences in disease states and use of these agents as monotherapy in PIMND versus as an adjunct therapy in RA and SLE. Nonetheless, ocrelizumab infusions in MS patients appear to be related to elevated risk of upper and lower respiratory infections, upper respiratory irritation during the infusions (typically the first infusion), peripheral herpes related infections, and some concern over malignancies, particularly breast cancers [25]. In terms of rituximab, such adverse events were not seen in our study or in prior studies. These differences in the profile of adverse events may highlight fundamental differences in the pharmacokinetic and pharmacodynamic properties of the two molecules, which remain to be explored. It is already known that the two molecules have differences in the mechanism whereby they achieve B-cell depletion. There may be important difference in tissue penetration, repletion rate, and secondary effects on T-cells. Same rationale is also relevant to other anti-CD 20 molecules that are under clinical development, such as ofatumumab and ublituximab. Each of theses may have a unique side effect profile related to their pharmacokinetic and pharmacodynamic properties.

It should also be noted that ocrelizumab was compared to Rebif, an interferon, in the OPERA I and II trials. Interferons are known to have anti-viral and anti-tumor activities. When comparing drug trials to interferons in general, this distinction needs to be kept in mind when assessing relative risk of infections or malignancies, which may be suppressed in the interferon treated cohort.

In summary, we report long-term safety of rituximab in PIMND. Rituximab was well tolerated over time. AE and SAEs remained low throughout the observation period. Patients remained clinically stable while receiving continuous rituximab infusions. Although this is small study, nevertheless, it makes important contributions to a number of growing data documenting the long-term tolerability and efficacy of rituximab as a viable option for the treatment of PIMND. Larger, prospective, multicenter studies are still needed to further corroborate long-term safety and tolerability of rituximab and other B-cell depleting agents in treating PIMND.

## Supporting information

S1 Fig Rituximab data excel sheet. (XLSX)
